# Multi-Wavelength PPG Amplitude Ratio Features for Cuffless Blood Pressure Estimation: An Exploratory Study

**DOI:** 10.3390/s26216897

**Published:** 2026-10-30

**Authors:** Hsiao-Huang Chang, Te-Min Lee, Shu-Lin Guo, Po-Lei Lee

**Affiliations:** 1 Department of Surgery, School of Medicine, Taipei Medical University, Taipei 110301, Taiwan; shchang@vghtpe.gov.tw; 2 Cardiovascular Center, Taipei Veterans General Hospital, Taipei 112201, Taiwan; 3 Department of Electrical Engineering, National Central University, Taoyuan 32001, Taiwan; temin.lee@gmail.com; 4 School of Medicine, Fu Jen Catholic University, New Taipei City 242062, Taiwan; 07908@cych.org.tw; 5 Department of Medical Research, Cathay General Hospital, Taipei 106324, Taiwan

**Keywords:** photoplethysmography (PPG), cuffless blood pressure estimation, amplitude ratio features, curvature-based feature detection

## Abstract

**Highlights:**

- A curvature-based procedure identifies PPG fiducial points across red, infrared, and green wavelengths.
- Green-light PPG yielded several of the features most strongly associated with cuff-based blood pressure in this cohort.
- Amplitude ratios involving the systolic peak and dicrotic notch showed stronger BP associations than most individual timing and amplitude features.
- Combined-feature linear regression models achieved cross-validated prediction errors of 12.87 mmHg for systolic BP and 7.17 mmHg for diastolic BP.
- This exploratory study supports further evaluation of multi-wavelength PPG features using larger cohorts and time-aligned BP references.

**Abstract:**

**Background:** Cuffless blood pressure (BP) estimation from photoplethysmography (PPG) remains challenging. This exploratory study investigated whether curvature-derived waveform features from multiple PPG wavelengths, particularly amplitude ratios, are associated with cuff-based BP readings. **Methods:** Red, infrared, and green PPG signals were recorded for 90 s from 48 physician-screened volunteers (23 males and 25 females; mean age, 63.41 ± 14.20 years). A single automated upper-arm cuff BP measurement was initiated approximately 2 s after each PPG recording. A curvature-based procedure identified pulse valleys, systolic peaks, dicrotic notches, and diastolic peaks, from which timing, amplitude, and amplitude ratio features were extracted. Separate linear regression models for systolic BP (SBP) and diastolic BP (DBP) were evaluated using participant-level ten-fold cross-validation. In each iteration, feature selection and model fitting were performed using only the nine training folds, and the fitted model was evaluated on the held-out fold. **Results:** In the descriptive analysis across all 48 participants, amplitude ratio features involving the systolic peak and dicrotic notch showed stronger associations with cuff-based BP readings than most individual timing and amplitude features. For green-light PPG, the systolic-peak-to-dicrotic-notch amplitude ratio (*Ratio_SN_*) had a correlation of approximately 0.56 with SBP and 0.48 with DBP. Based on out-of-fold predictions, the best combined-feature models achieved root mean square errors of 12.87 mmHg for SBP and 7.17 mmHg for DBP, with corresponding *R*^2^ values of 0.87 and 0.83. **Conclusions:** Multi-wavelength PPG amplitude ratio features, particularly those involving the dicrotic notch, warrant further investigation for cuffless BP estimation. The substantial SBP error, small cohort, and use of a single cuff measurement obtained after each PPG recording limit interpretation. These findings do not establish clinical measurement accuracy or continuous BP tracking.

## 1. Introduction

Hypertension is one of the most prevalent and severe chronic diseases worldwide. Persistently elevated blood pressure substantially increases the risk of stroke, myocardial infarction, chronic kidney disease, and retinopathy [1]. Continuous and convenient blood pressure (BP) monitoring therefore holds great clinical value for early diagnosis and long-term health management. The most commonly used device in both clinical and home settings remains the cuff-based sphygmomanometer, which estimates systolic blood pressure (SBP) and diastolic blood pressure (DBP) by occluding the brachial artery with an inflatable cuff and detecting either Korotkoff sounds or oscillometric signals. Although cuff-based devices are well established and generally accurate, they still present several drawbacks: (1) measurements require a fixed posture and cannot provide continuous monitoring; (2) the inflating cuff induces compression and discomfort, making long-term use impractical; (3) dynamic BP variations during daily activities cannot be captured; and (4) the relatively bulky device design limits its applicability in wearable healthcare [2]. Furthermore, their accuracy can be affected by inappropriate cuff size, obesity, or muscle mass. Consequently, in the context of an aging society and the increasing prevalence of chronic diseases, conventional cuff-based sphygmomanometers are gradually becoming insufficient for long-term and real-time BP monitoring.

To overcome these limitations, researchers have explored cuffless, continuous BP monitoring methods. Among these, Pulse Arrival Time (PAT) and Pulse Transit Time (PTT) have attracted considerable attention in recent years. PAT is defined as the time interval between the R-wave of the electrocardiogram (ECG) and the onset of the photoplethysmography (PPG) waveform [3]. PTT, in contrast, is the time required for the pulse wave to propagate between two arterial sites, typically measured as the delay between systolic peaks at two peripheral PPG locations. Numerous studies have demonstrated strong correlations between PAT/PTT and BP changes, highlighting their potential for non-invasive and continuous BP assessment [4]. However, both methods face practical limitations. For PAT, (1) simultaneous acquisition of ECG and PPG is required, necessitating two separate sensing modules; (2) ECG lead placement often spans opposite sides of the body (e.g., chest and wrist), which reduces convenience for wearable applications [5]. For PTT, the need to acquire PPG signals from different body sites introduces susceptibility to motion artifacts [6]. Sensor modules may experience inconsistent disturbances, and physiological variability such as heart rate and BP fluctuations can further distort the inter-signal time delay, thereby reducing accuracy—particularly during physical activity. Thus, despite their theoretical potential, PAT and PTT require multi-sensor integration across distant measurement sites and are easily influenced by inter-individual variability, limiting their widespread application.

Recent PPG-only studies have further explored the BP-related information contained in pulse-wave morphology. Liu et al. (2025) used smartphone PPG waveform analysis and interpretable machine learning for BP estimation [7]. Lin et al. (2026) examined waveform amplitude, contour sharpness, and harmonic features for interpretable assessment of systolic BP status [8]. These studies illustrate the range of morphological information available from PPG signals. In contrast to approaches based on a single optical channel, the present study compares red, infrared, and green fingertip PPG and examines amplitude ratios formed from curvature-detected fiducial points.

BP estimation using PPG alone has developed along two complementary directions. Some studies analyze the full PPG waveform using deep learning, allowing predictive patterns to be learned without prespecifying individual fiducial points. For example, Moulaeifard et al. (2025) benchmarked models trained directly on PPG waveforms and showed that performance may deteriorate when evaluated on external datasets [9]. Other studies extract explicitly defined features from the pulse contour. Earlier approaches used waveform area and the second derivative of PPG (SDPPG) to characterize pulse morphology and vascular properties [10,11]. More recently, Liu et al. (2025) investigated smartphone PPG waveform features for BP estimation [7], while Lin et al. (2026) examined amplitude, contour sharpness, and harmonic features for interpretable classification of SBP status [8]. These studies demonstrate that both learned waveform representations and defined morphological features may contain BP-related information, although their outcomes and validation methods differ [12].

The present study focuses on a specific question that remains distinct from full-waveform modeling: whether the relative amplitudes of the systolic peak, dicrotic notch, and diastolic peak provide useful information for SBP and DBP estimation across red, infrared, and green fingertip PPG signals acquired with the same device. We use curvature to locate these fiducial points and compare timing, absolute-amplitude, and within-waveform amplitude ratio features. Neither the curvature formula nor amplitude ratios are claimed as new mathematical concepts; the contribution is the extraction and comparative assessment of these interpretable features across three wavelengths in this exploratory cohort.

In addition, the wavelength of incident light critically influences PPG signals. Variations in absorption coefficients of blood components (e.g., oxy- and deoxyhemoglobin, glucose, water, lipids) [13], skin transmittance, and environmental factors all affect waveform morphology. Compared to the red (660 nm) and near-infrared (940 nm) wavelengths commonly used for SpO_2_ measurement, green light (520 nm) exhibits nearly identical absorption for oxy- and deoxyhemoglobin, thereby capturing overall hemodynamic fluctuations more effectively. This property makes green light especially useful in wearable devices for heart rate monitoring. However, major PPG-BP databases such as MIMIC-II/III/IV, UCI/Kaggle PPG-BP Database, BIDMC PPG & Respiration, and VitalDB (see Table 1) predominantly employ red and near-infrared wavelengths, often with relatively low sampling rates (<250 Hz). Therefore, the second objective of this study is to incorporate green-light PPG as a source for cuffless BP estimation and to compare its performance against red (660 nm) and near-infrared (940 nm) signals. By systematically evaluating BP estimation accuracy across these three wavelengths, we aim to compare the associations and BP estimation results of features extracted from the three wavelengths in this cohort.

The remainder of this paper is organized as follows. Section 2 describes the measurement system, subject recruitment, and the curvature-based method for PPG feature extraction, including the definition of timing, amplitude, and amplitude ratio features. Section 3 presents the experimental results, including correlation analysis and regression-based blood pressure estimation. Section 4 provides a detailed discussion of the findings, highlighting the importance of amplitude ratio features and the comparative performance of different wavelengths. Finally, Section 5 concludes the study and outlines future directions for the further evaluation of PPG-based blood pressure estimation.

## 2. Materials and Methods

### 2.1. System Architecture

**Figure 1:**
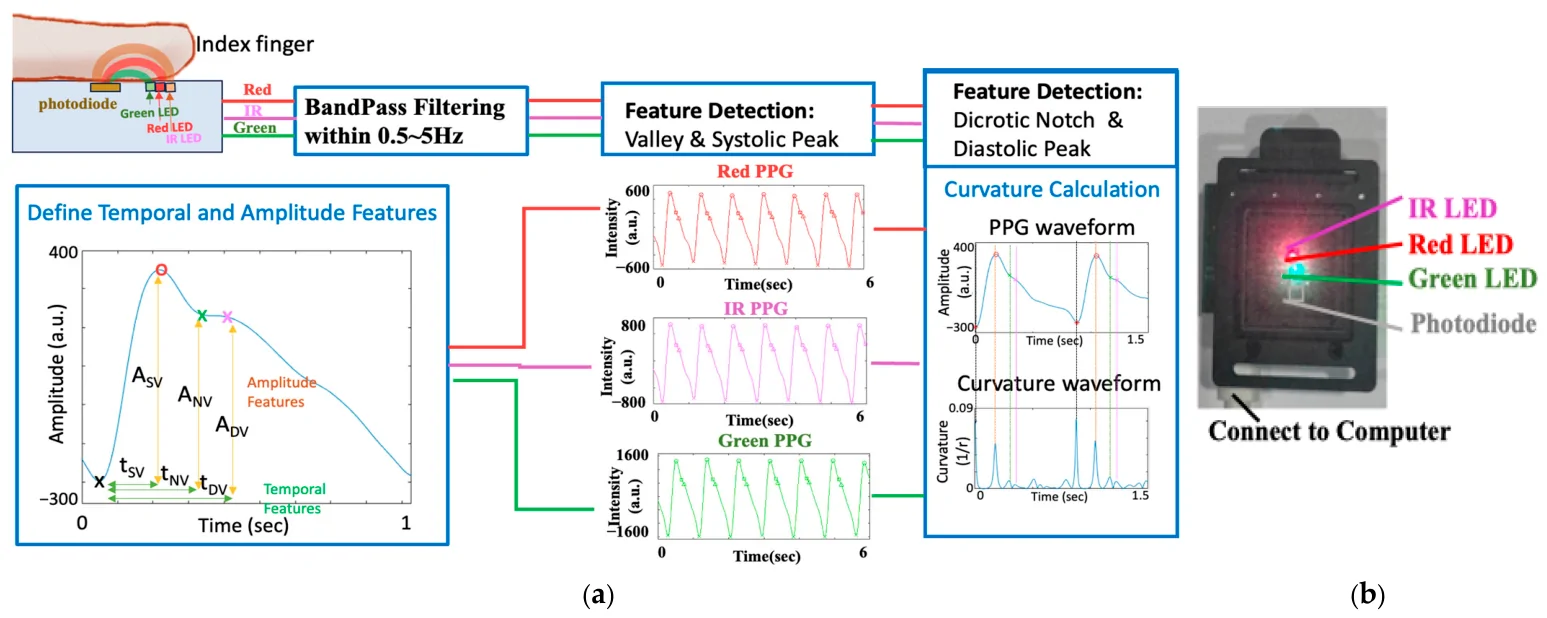
Overview of the three-wavelength non-invasive reflectance-mode PPG measurement system used in this study. (**a**) Signal processing workflow illustrating bandpass filtering, cardiac cycle segmentation using the curvature-based method, and detection of fiducial points (valley, systolic peak, dicrotic notch, and diastolic peak) from each wavelength for subsequent cuffless blood pressure estimation. (**b**) Photograph of the three-wavelength reflectance-mode PPG measurement device.

Figure 1a illustrates the non-invasive, reflective-type PPG measurement device (Model: OCS112; Taiwan tBPC Co., Hsinchu, Taiwan) used in this study. The device integrates three light sources: a 660 nm red LED, a 940 nm infrared light-emitting diode (LED), and a 520 nm green LED. This system has been certified as a medical device by the Taiwan Food and Drug Administration (TFDA, certificate number: 005860). This certification concerns the PPG acquisition device and does not constitute clinical validation of the BP estimation model evaluated here. The device adopts a diffractive optical element (DOE)-based reflective sensing technology, which focuses the emitted light into a narrow half-angle of approximately 20°, thereby improving optical efficiency. The three light sources are sequentially activated at a frequency of 500 Hz, ensuring that PPG signals across all wavelengths are acquired synchronously within a single cardiac cycle. This reflective PPG system is designed for fingertip measurement, with both light sources and photodetectors placed on the same side. Blood volume changes and optical absorption characteristics are captured through the reflected light. The raw PPG signals were preprocessed using a band-pass filter with a passband of 0.5–5 Hz to attenuate baseline variation and higher-frequency noise before feature extraction. This preprocessing step was separate from the centered five-sample Savitzky–Golay derivative estimation used for curvature calculation, as described in Section 2.3. Fiducial points are then extracted from each wavelength signal. Specifically, the curvature values of the PPG signals are computed to segment cardiac cycles, and key fiducial points—including valley, systolic peak, dicrotic notch, and diastolic peak—are identified. Their corresponding amplitudes, amplitude ratios, and temporal intervals are further calculated as signal features, which are subsequently used for cuffless blood pressure estimation of both systolic and diastolic pressures (see below). Figure 1b shows the optical configuration of the reflective PPG device, including the arrangement of the three LEDs and the photodetector.

### 2.2. Subjects and Experimental Design

A physician screened the prospective participants for eligibility before they were referred to the research team. Individuals with a known history of arrhythmia, valvular heart disease, coronary artery disease, myocardial infarction, cardiomyopathy (including virus-associated cardiomyopathy), or functional cardiac disorders were not included. The final study cohort comprised 48 clinical volunteers (23 males and 25 females; mean age: 63.41 ± 14.20 years). Their mean systolic blood pressure (SBP) and diastolic blood pressure (DBP) were 138.18 ± 19.23 mmHg and 81.96 ± 11.17 mmHg, respectively. Four participants had both SBP > 140 mmHg and DBP > 90 mmHg. The demographic and BP characteristics of the 48 participants are summarized in Table 2. Participant recruitment was not designed to satisfy the sample-size or predefined BP-distribution requirements of a standardized clinical BP-device validation protocol. The cohort was used to explore associations between multi-wavelength PPG features and cuff-based BP readings.

**Figure 2:**
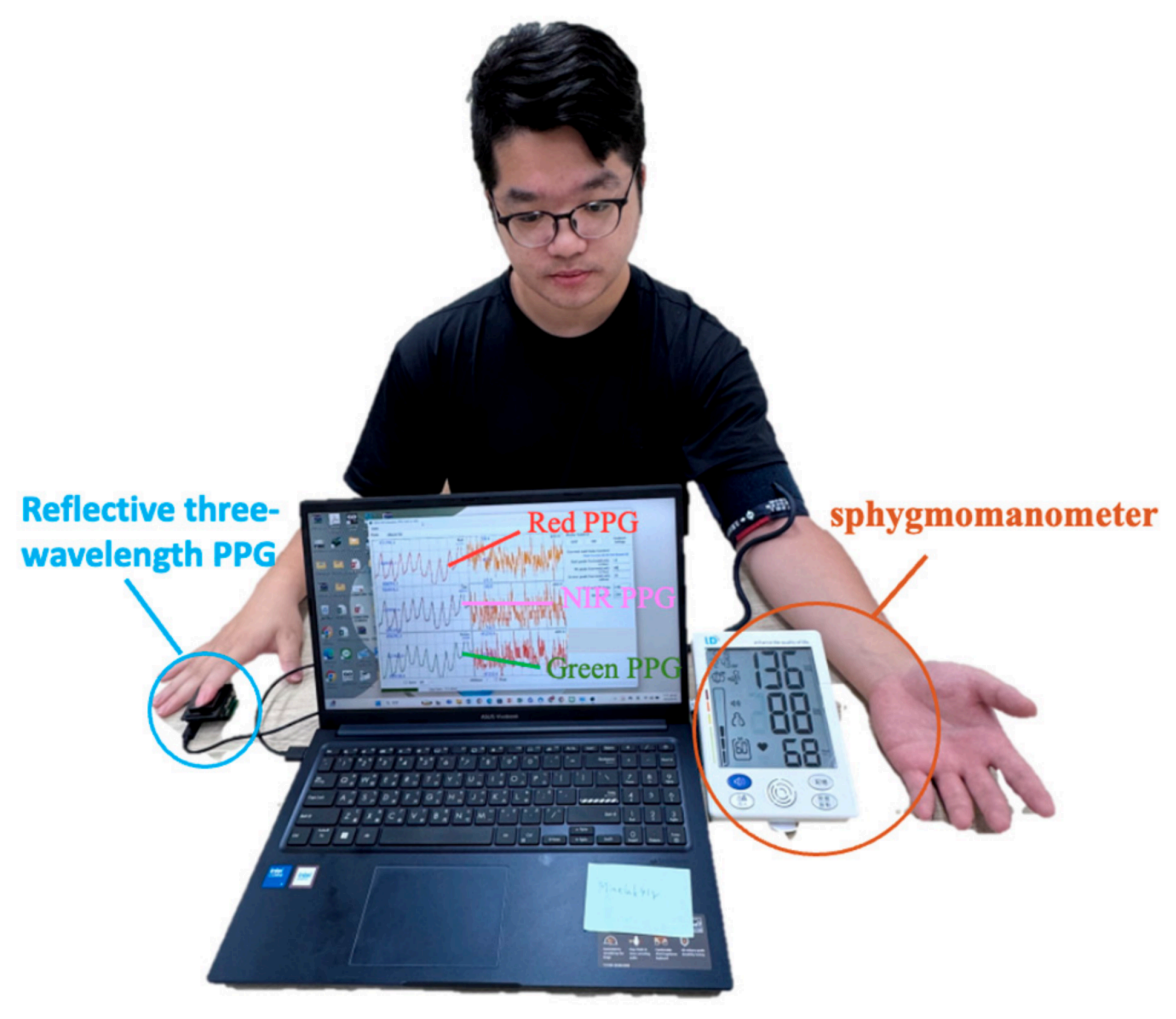
Experimental setup for blood pressure measurement. The subject was seated with the right index finger placed in the reflective three-wavelength PPG sensor (red, infrared, and green channels), while a reference sphygmomanometer (Omron HEM-907, Kyoto, Japan) was applied to the left upper arm for cuff-based BP measurement.

Measurements were performed in a quiet environment while participants sat comfortably in chairs with armrests. The PPG sensor was placed on the right index finger, and an upper-arm cuff connected to an Omron HEM-907 sphygmomanometer (Omron Healthcare Co., Kyoto, Japan) was placed on the left arm. Thus, the PPG sensor and cuff were positioned on contralateral limbs (Figure 2). After a 30 s resting period and confirmation that discernible PPG waveforms were present at all three wavelengths, PPG signals were recorded for 90 s. Participants were instructed to remain still during PPG acquisition. Cuff BP measurement was initiated approximately 2 s after the end of the PPG recording. During the interval and subsequent cuff measurement, participants maintained the same seated posture as during PPG acquisition. Throughout the procedure, they were instructed to remain still and refrain from speaking, with both arms resting comfortably on the chair’s armrests. A single cuff measurement provided the SBP and DBP values used for model development and evaluation. These values were obtained from the automated cuff device; auscultatory reference measurements by two qualified observers were not performed. PPG acquisition and cuff BP measurement were therefore sequential rather than simultaneous, and the cuff reading was used as an intermittent comparison value for the exploratory analysis.

For PPG feature extraction, the analysis was limited to participants with a resting heart rate between 30 and 100 beats/min. Within each participant’s recording, cardiac cycles whose duration differed by more than 10% from that participant’s median cycle duration were excluded before feature extraction to reduce the influence of irregular or incorrectly detected cycles. This criterion was applied to individual cardiac cycles. The study protocol was approved by the Institutional Review Board of Landseed International Hospital, Taoyuan, Taiwan (LSIHIRB: 21-003-A2), and all procedures were conducted in accordance with the approved protocol.

### 2.3. PPG Feature Point Detection

In this study, we propose using a curvature-based analysis method to detect turning points in the PPG waveform, thereby accurately identifying key fiducial points such as the systolic peak, dicrotic notch, and diastolic peak. Compared with the conventional second-derivative method [10,14], the curvature method estimates curvature using an appropriate local window (five data points in this study, sampled at 500 Hz, corresponding to a 10 ms window).

For a one-dimensional PPG signal *x*(*t*), the discrete time sequence around point *t_i_* is denoted as *X* = {*x_i_*_−2,_*x_i_*_−1,_*x_i_*_,_*x_i_*_+1,_*x_i_*_+2,_}. The first and second derivatives at *t_i_* are approximated as follows:

(1) $$x^{\prime }(t_{i})\approx \frac{-x_{i+2}+8x_{i+1}+0x_{i}-8x_{i-1}+x_{i-2}}{12∆t}$$

and

(2) $$x^{\prime \prime }(t_{i})\approx \frac{-x_{i+2}+16x_{i+1}-30x_{i}+16x_{i-1}-x_{i-2}}{12{∆t}^{2}}$$

where *x*^′^(*t_i_*) and *x*^″^(*t_i_*) represent the first and second derivatives of the PPG signals *x*(*t*) at time point *t_i_*. The curvature *κ*(*t_i_*) at these five points can therefore be calculated as follows:

(3) $$\kappa \left(t_{i}\right)=\frac{\left|x^{\prime \prime }\left(t_{i}\right)\right|}{{\left(1+{\left[x^{\prime }\left(t_{i}\right)\right]}^{2}\right)}^{3/2}}$$

In Equations (1) and (2), the derivatives were estimated using a centered five-sample, fourth-order Savitzky–Golay (SG) formulation. Each derivative estimate was assigned to the center sample of the window, so the derivative-estimation step did not introduce an additional shift in the reported time index. With five samples and a fourth-order polynomial, this formulation is equivalent to a five-point finite-difference derivative estimate and does not provide additional smoothing. The SG derivative calculation was applied after the 0.5–5 Hz band-pass preprocessing described in Section 2.1. Other approaches, including forward–backward Butterworth or Chebyshev filtering followed by derivative estimation, could also be used to prepare the PPG signal for curvature calculation. These alternatives were not compared in the present study, and their effects on waveform morphology and fiducial-point timing remain to be evaluated.

The resulting first- and second-derivative estimates were substituted into Equation (3) to calculate curvature. The curvature method calculates the reciprocal of the radius *R* of the osculating circle formed by the input points. Specifically, the curvature *κ* incorporates the second derivative |*x*^″^(*t*)| normalized by ${\left(1+{\left[x^{\prime }\left(t\right)\right]}^{2}\right)}^{3/2}$, thereby reducing susceptibility to amplitude variations, gain differences, or temporal jitter across subjects. This is in contrast to approaches that analyze the first- or second-derivative waveform alone—the velocity photoplethysmogram (VPG) and acceleration photoplethysmogram (APG) [15], respectively. The curvature calculation combines both derivatives according to the conventional curvature formula. Local curvature extrema were then used as candidates for PPG fiducial points.

**Figure 3:**
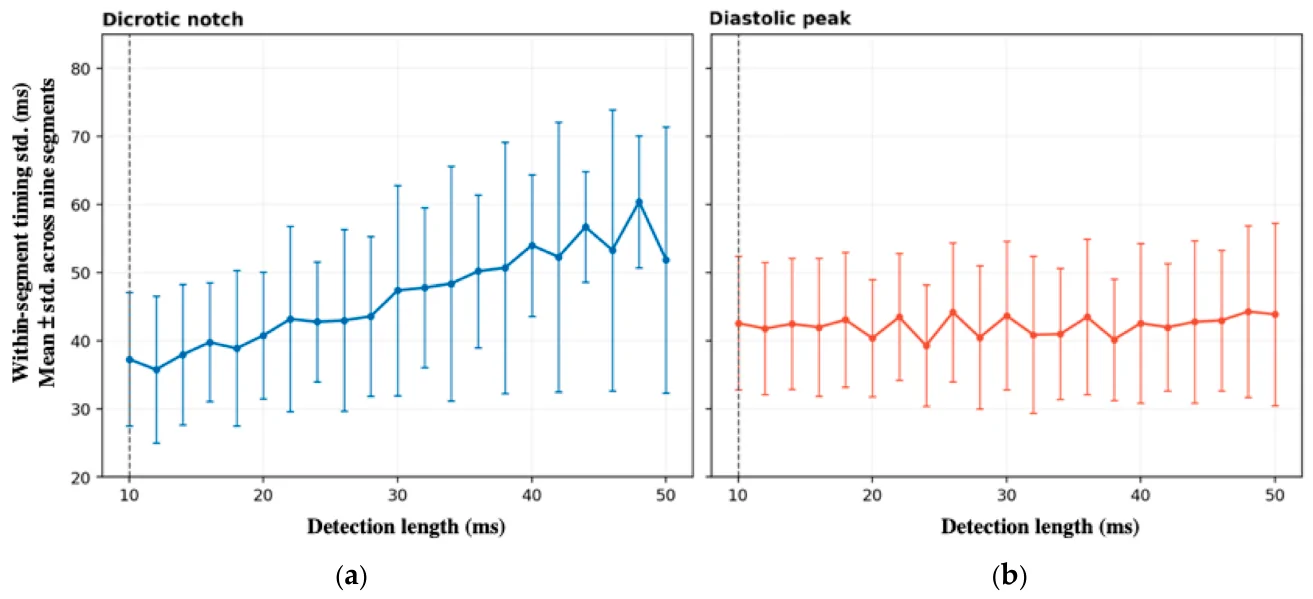
Preliminary sensitivity analysis of curvature detection length. A 90 s PPG recording from one separate healthy volunteer (mean heart rate: 66.74 beats/min) was divided into nine non-overlapping 10 s segments. Within each segment, the standard deviation of the valley-to-fiducial-point time interval was calculated across cardiac cycles. Points represent the mean of the nine segment-level standard deviations; error bars represent their standard deviation. (**a**) Valley-to-dicrotic-notch interval. (**b**) Valley-to-diastolic-peak interval. Detection lengths ranged from 0.010 to 0.050 s in increments of 0.002 s. The dashed line marks the setting used in the main analysis.

To examine the choice of curvature detection length, a preliminary sensitivity analysis was performed using a 90 s PPG recording from a separate healthy volunteer who was not included in the 48-participant BP estimation cohort. The volunteer’s mean heart rate was 66.74 beats/min. The recording was divided into nine non-overlapping 10 s segments. For each cardiac cycle, the intervals from the pulse valley to the detected dicrotic notch and diastolic peak were calculated. At each detection-length setting, the standard deviation of each interval was calculated across cardiac cycles within each 10 s segment. The mean and standard deviation of the nine segment-level values are presented in Figure 3. Detection length was varied from 0.010 to 0.050 s in increments of 0.002 s, corresponding to settings of 5 to 25 samples at the 500 Hz sampling rate. The mean within-segment variability of dicrotic-notch timing generally increased with detection length, whereas diastolic-peak timing varied less across the tested settings. We therefore retained the shortest tested setting, 0.010 s, for subsequent feature extraction.

**Figure 4:**
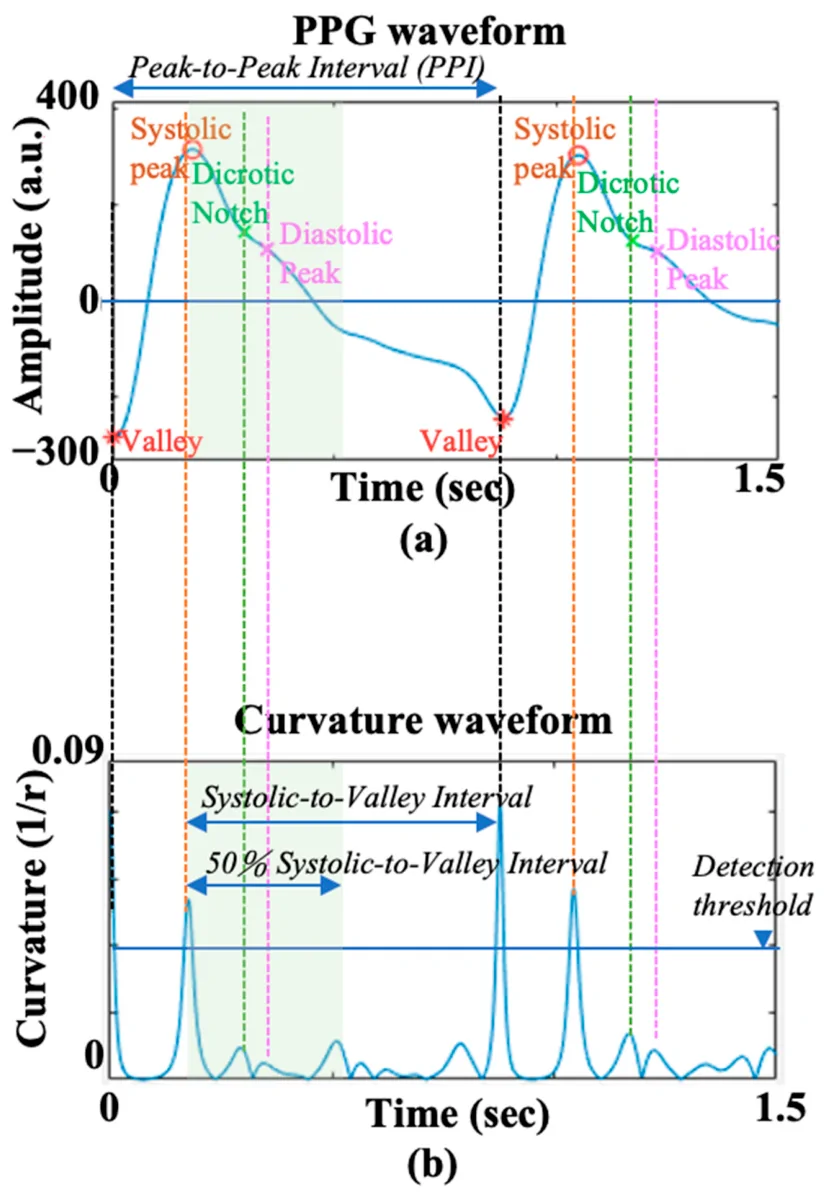
Example of PPG waveform and corresponding curvature waveform. (**a**) PPG signal showing the detected feature points: valley, systolic peak, dicrotic notch, and diastolic peak, along with the peak-to-peak interval (PPI). (**b**) Curvature waveform derived from the PPG signal, illustrating the detection threshold, systolic-to-valley interval, and 50% systolic-to-valley interval used for identifying feature points.

As shown in Figure 4, the PPG waveform and its corresponding curvature over two cardiac cycles demonstrate that valleys and systolic peaks consistently exhibit the highest curvature values, with valleys typically producing greater curvature than systolic peaks. The subsequent two curvature peaks correspond to the dicrotic notch and the diastolic peak. The PPG feature detection procedure in this study is summarized as follows:

(a) Identify all curvature peaks from the computed curvature waveform.
(b) A half-maximum threshold, defined as 50% of the maximum curvature peak value, is applied to retain prominent curvature peaks as candidate valleys and systolic peaks. This relative threshold provides a simple amplitude-normalized criterion: peaks below half of the reference maximum are rejected, whereas peaks at or above it are retained for the subsequent temporal and morphological checks. Half-maximum criteria are commonly used to define transitions in measured waveforms, and relative peak-amplitude thresholds have also been applied in PPG fiducial-point detection [16]. Here, 50% is used as an operational candidate-selection threshold, rather than assumed to be a universally optimal value.
(c) Based on [17], the normal duration of left ventricular systole at rest (from mitral valve closure to aortic valve closure) is typically 270–372 ms. Accordingly, for two consecutive curvature peaks above the threshold, the first is designated as the valley and the second as the systolic peak. The interval between two valleys is defined as the peak-to-peak interval (PPI).
(d) Within the systolic-to-valley interval, the first 50% of the segment is examined for two additional curvature peaks, which are assigned as the dicrotic notch and diastolic peak.
(e) Valleys, systolic peaks, dicrotic notches, and diastolic peaks were detected separately in the red, infrared, and green PPG signals. Previous multi-wavelength PPG studies have reported wavelength-dependent differences in pulse-feature timing [18]. For cross-wavelength systolic-peak matching, Huang et al. used a tolerance of 5% of the interbeat interval (IBI), which corresponds to 100 ms at a heart rate of 30 beats/min and 30 ms at 100 beats/min [19]. In the present study, we used a fixed upper tolerance of 100 ms to match corresponding fiducial points across the three wavelengths. Cardiac cycles with corresponding points outside this tolerance were excluded from multi-wavelength feature analysis.

For each cardiac cycle and wavelength, we calculated the amplitude differences from the pulse valley to the systolic peak, dicrotic notch, and diastolic peak. These amplitudes were then used to derive within-wavelength amplitude ratios, including the systolic-peak-to-dicrotic-notch ratio (*Ratio_SN_*) and systolic-peak-to-diastolic-peak ratio (*Ratio_SD_*), as input features for the subsequent blood pressure estimation models.

In this study, curvature analysis was employed to identify stable feature points on the PPG waveform. As illustrated in Figure 4, the curvature is defined as *κ* = 1/R, where R is the local radius of curvature. For example, in the *i*th PPG pulse wave, PPG*_i_*, the two most prominent inflection points located between the systolic peak and the subsequent valley exhibited curvature values of *κ*_1_ = 0.0154 and *κ*_2_ = 0.0049, corresponding to radii of R_1_ = 64.98 and R_2_ = 200.64, respectively. In the *i* + 2*nd* PPG pulse wave PPG*_i_*_+2_, the two clear inflection points between the systolic peak and the next valley showed curvature values of *κ*_3_ = 0.0297 and *κ*_4_ = 0.0206, with corresponding radii of R_3_ = 33.71 and R_4_ = 43.55. These results demonstrate that physiologically meaningful points—such as the systolic peak, dicrotic notch, and diastolic descent—consistently coincide with local curvature extrema, independent of amplitude fluctuations.

### 2.4. Definition of PPG Features

Previous PPG-based BP studies have extracted several types of interpretable waveform features. Timing features include pulse duration, systolic and diastolic intervals, and delays associated with reflected waves; morphological features include systolic and diastolic areas, peak amplitudes, and ratios between waveform components [1,11,20]. Shin and Min examined features related to PPG pulse morphology and reflected-wave timing, while Hasanzadeh et al. identified the dicrotic-notch reflection index, together with other PPG-derived measures, as informative for BP estimation [1,20]. More recently, Liu et al. investigated timing, amplitude-difference, area, and curvature-related waveform indicators from smartphone PPG and assessed their contributions to BP prediction [7,20]. These studies motivate the examination of features that describe both the positions and relative amplitudes of landmarks within the PPG pulse.

Accordingly, we extracted timing intervals and valley-referenced amplitudes for the systolic peak, dicrotic notch, and diastolic peak in each wavelength channel. We also calculated within-waveform amplitude ratios involving the systolic peak and either the dicrotic notch or diastolic peak. Ratios were examined because prior work has found reflected-wave and normalized morphological features informative for BP estimation [1,7,21]; in the present study, their predictive contribution relative to absolute timing and amplitude features is evaluated empirically rather than assumed in advance.

In this study, PPG feature extraction was based on the prior literature, incorporating area-based features, timing features of characteristic points, and amplitude-based features.

Physiologically, the systolic rising edge in the PPG waveform reflects the expansion of the arterial system due to blood inflow, with the rate of expansion associated with cardiac contractility and the systolic peak amplitude linked to stroke volume. The dicrotic notch and diastolic peak are generated by wave reflections, with their timing and location influenced by arterial stiffness. Finally, the diastolic decay corresponds to arterial contraction during blood outflow, primarily determined by vascular resistance and compliance.

**Figure 5:**
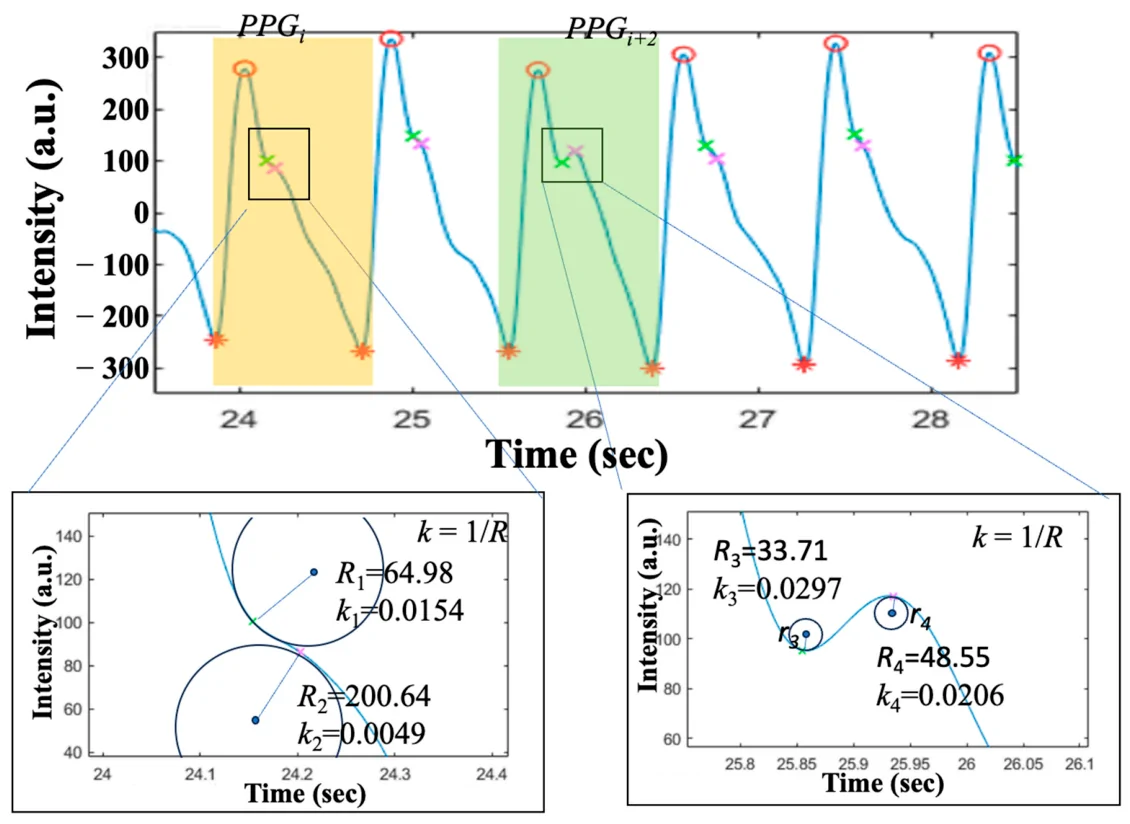
Example PPG waveform and corresponding curvature analysis across two cardiac cycles. The asterisk (*), red circle (○), green cross (×), and pink cross (×) markers indicate the valley, systolic peak, dicrotic notch, and diastolic peak, respectively. These fiducial points are identified based on distinct curvature extrema in the PPG waveform.

Considering these physiological mechanisms, this study focuses exclusively on the timing and amplitude features of four primary PPG landmarks—valley, systolic peak, dicrotic notch, and diastolic peak—for cuffless blood pressure estimation. Figure 5 illustrates a representative PPG pulse wave along with the extracted timing and amplitude features. According to [22], the systolic peak corresponds to the forward pressure wave generated by left ventricular contraction, the dicrotic notch reflects the closure of the aortic valve, and the diastolic peak arises from the peripheral reflected wave [7]. The dicrotic notch represents a turning point in the PPG waveform, marking the moment when the heart ceases to actively propel blood while the reflected wave from the peripheral vasculature has not yet returned to the measurement site. This inflection point reflects the static pressure within the artery, which serves as an important physiological reference for estimating both systolic and diastolic pressures.

**Figure 6:**
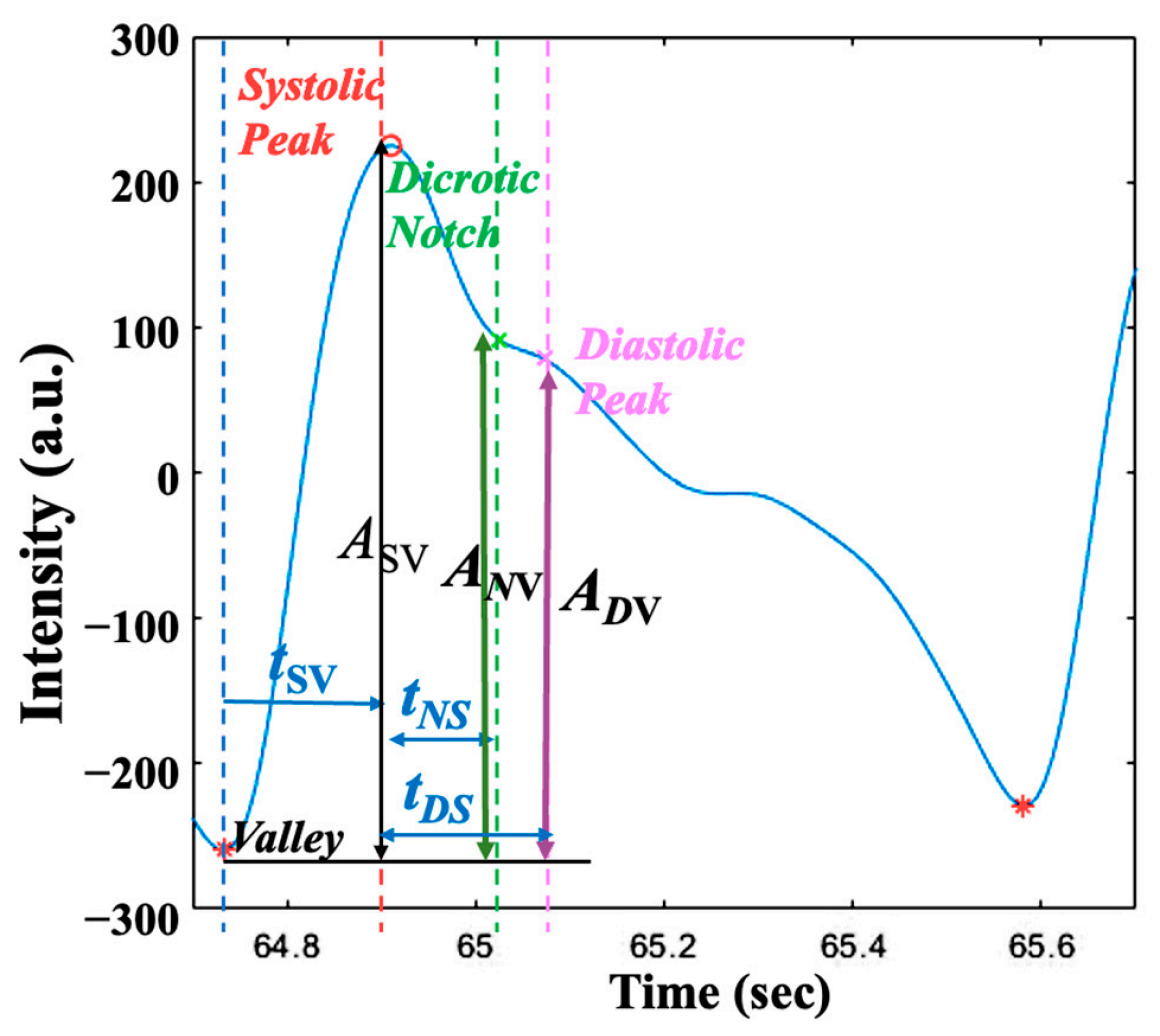
Definition of timing and amplitude features extracted from a single PPG pulse wave. Feature points include valley (V), systolic peak (S), dicrotic notch (N), and diastolic peak (D). Temporal intervals (*t_SV_*, *t_NS_*, *t_DS_*) and amplitude differences (*Amp_SV_*, *Amp_NV_, Amp_DV_*) are illustrated.

Based on the four identified fiducial points, timing and amplitude features were extracted to characterize the morphological properties of each PPG pulse. As illustrated in Figure 6, three timing intervals (*t_SV_*, *t_NS_*, and *t_DS_*) and three amplitude differences (AmpSV, AmpNV, and AmpDV) were defined using the valley, systolic peak, dicrotic notch, and diastolic peak as reference points. As summarized in Table 3, this study defines three categories of PPG-derived features:

(a) Timing Features
  ▯ *t_sv_*__light_: time interval from systolic peak to valley under the specified light source (i.e., red, infrared, or green).
  ▯ *t_NS_*__light_: time interval from dicrotic notch to systolic peak under the specified light source.
  ▯ *t_DS_*__light_: time interval from diastolic peak to systolic peak under the specified light source.
(b) Amplitude Features
  ▯ *A_SV_*__light_: amplitude difference between systolic peak and valley under the specified light source.
  ▯ *A_NV_*__light_: amplitude difference between dicrotic notch and valley under the specified light source.
  ▯ *A_DV_*__light_: amplitude difference between diastolic peak and valley under the specified light source.
(c) Amplitude Ratio Features
  ▯ *Ratio_SN_*_light = ratio of *A_SV_*__light_ to _ANV_light_ under the specified light source.
  ▯ *Ratio_SD_*_light = ratio of *A_SV_*__light_ to *A_DV_*__light_ under the specified light source.

### 2.5. Univariate Correlation Analysis of PPG Features and Blood Pressure

**Figure 7:**
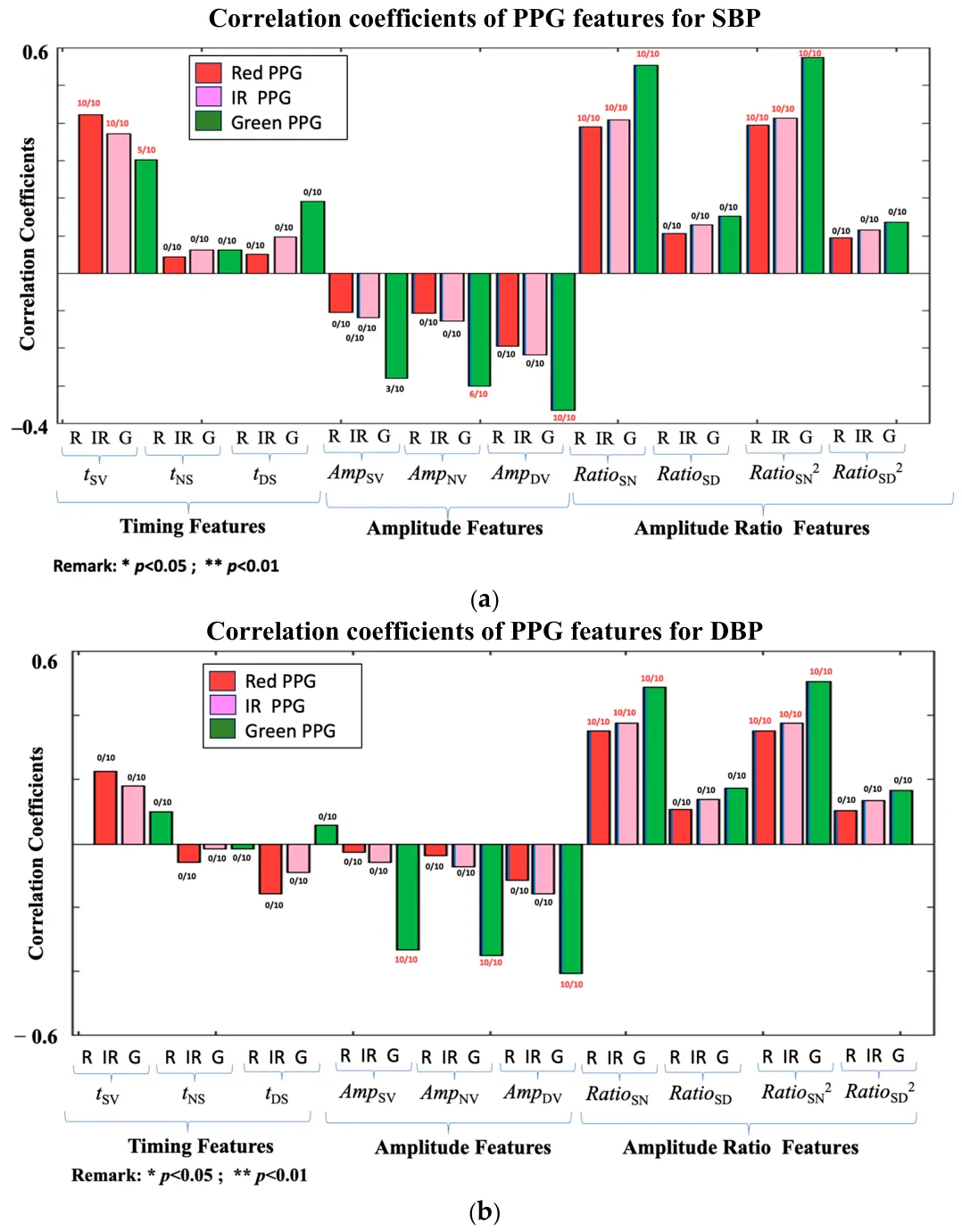
Pearson correlation coefficients and cross-validation feature-selection frequencies for 30 PPG-derived features. Bar heights show the correlations with cuff-based blood pressure measurements across all 48 participants: (**a**) systolic blood pressure (SBP) and (**b**) diastolic blood pressure (DBP). Labels above or below the bars indicate the number of ten-fold cross-validation iterations in which a feature met the Pearson correlation criterion of *p* < 0.05, calculated using only the nine training folds in that iteration. The full-cohort correlation coefficients shown by the bars were used for descriptive analysis and were not used to select features for the cross-validated models. Features selected in at least five of the ten iterations (≥5/10) (marked in red) were designated as frequently selected features. This designation summarizes selection frequency; feature selection for model fitting was performed independently within the training folds of each iteration.

We examined each of the 30 candidate PPG features for its association with cuff-based SBP and DBP. For each feature–BP pair, Pearson’s correlation coefficient (r) and its two-sided *p* value were calculated using MATLAB R2024b (MathWorks, Natick, MA, USA). Figure 7 displays the correlation coefficients calculated across all 48 participants for descriptive purposes. It also shows how often each feature met the *p* < 0.05 selection criterion across the ten cross-validation iterations. In each iteration, the selection test was performed using only the nine training folds; the full-cohort correlation coefficients displayed as bar heights were not used to select features for the cross-validated models. Feature selection for cross-validated BP estimation was performed separately within the training data of each fold, as described in Section 2.6.

By analyzing significance of correlation coefficient for each PPG feature, we identified which PPG features (e.g., systolic upstroke time, amplitude ratios, and dicrotic notch indices) provide the greatest predictive value for systolic and diastolic pressures. The two-sided *p* value of each feature–BP correlation was used as the feature-selection criterion (*p* < 0.05) within the nine training folds of each cross-validation iteration. This univariate criterion identifies features associated with BP in the training data but does not, by itself, establish their physiological relevance or account for correlations among features. For *n* subjects in the dataset being analyzed, the Pearson correlation coefficient between the *k*th PPG feature and SBP or DBP was calculated as follows:

(4) $$r=\frac{\sum_{i=1}^{n}\left(f_{i}-\bar{f}\right)\left(y_{i}-\bar{y}\right)}{\sqrt{\sum_{i=1}^{n}{\left(f_{i}-\bar{f}\right)}^{2}}\cdot \sqrt{\sum_{i=1}^{n}{\left(y_{i}-\bar{y}\right)}^{2}}},$$

where *f_i_* is the *kth* PPG feature from *i*th subject, $\bar{f}$ is the averaged value of *f_i_* from all subjects, *y_i_* is the SBP or DBP of *i*th subject measured from sphygmomanometer, $\bar{y}$ is the average of *y_i_* from all subjects, and *r* is the correlation coefficient between *f* and *y*.

To test the statistical significance of the correlation value, the following *t*-test was performed:

(5) $$T=r\sqrt{\frac{n-2}{1-r^{2}}},$$

where *T* is the *t*-test value with n − 2 degrees of freedom, and *r* is the value of calculated correlation coefficient. From this *t*-test, the *p*-value representing the statistical confidence of correlation value *r* can be obtained.

### 2.6. Linear Regression Analysis of PPG Features and Blood Pressure Prediction

We evaluated the 30 PPG-derived features listed in Table 3 for estimating systolic blood pressure (SBP) and diastolic blood pressure (DBP). Separate linear regression models were developed for the two outcomes. We first compared models using predefined groups of timing, amplitude, and amplitude ratio features from each wavelength. We then evaluated selected-feature models combining the three wavelengths or using green-light features alone. For either BP outcome, the regression model was expressed as:

*Y* = *β*_0_ + *β*_1_∙*f*_1_ + *β*_2_∙*f*_2_+…+*β_m_*∙*f_m_* + *ε*, (6)

where the *f*_1_, *f*_2_, …, *f_m_* are *m* features selected from the thirty features in Table 3, *β*_0_ is the intercept, *β*_1_, *β*_2_, …, *β_m_* are the regression coefficients corresponding to the m features, and ε represents the residue between the linear regression output *Y* and the blood pressure. Values were obtained using the sphygmomanometer.

Model performance was evaluated using participant-level ten-fold cross-validation. The 48 participants were randomly assigned to ten non-overlapping folds: eight folds contained five participants each, and two contained four participants each. Assignment was not stratified by age, sex, or BP level, and all data from a participant were kept in the same fold. In each iteration, one fold was held out for testing and the remaining nine folds were used for training. For the selected-feature models, Pearson’s *r* and its two-sided *p* value were calculated between each candidate feature and the reference BP measurements using only the training participants. Features with *p* < 0.05 were selected separately for SBP and DBP. A new regression model was fitted using the selected training features and then applied to the held-out participants. Thus, the selected feature set could differ between iterations.

**Figure 8:**
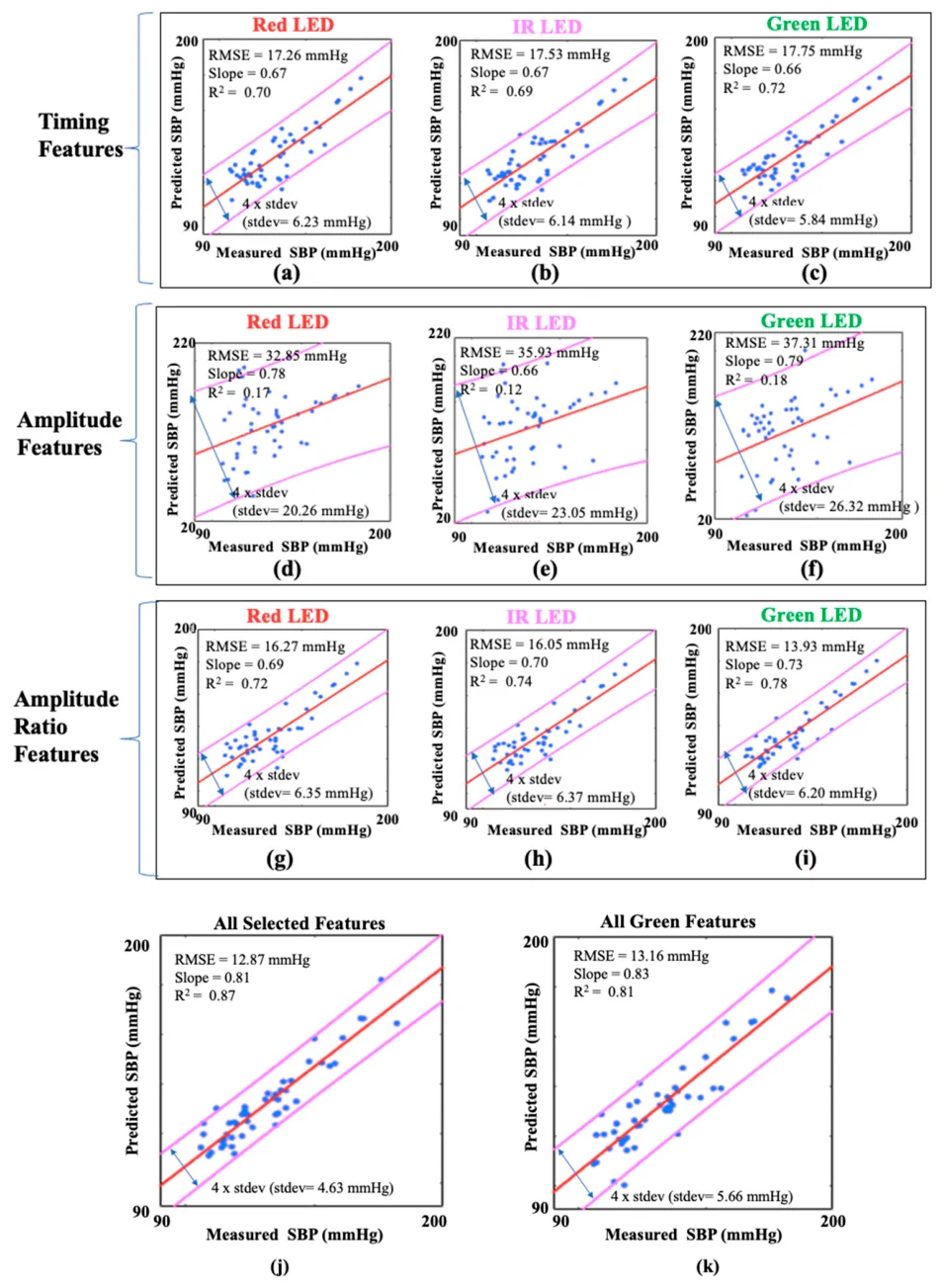
Systolic blood pressure (SBP) estimation using different PPG feature categories with participant-level ten-fold cross-validation (48 participants). (**a**–**c**) Timing features from red, infrared, and green PPG, respectively; (**d**–**f**) amplitude features from the three wavelengths; and (**g**–**i**) amplitude ratio features from the three wavelengths. (**j**) Features selected from all three wavelengths using only the nine training folds in each iteration (*p* < 0.05). (**k**) Corresponding feature selection restricted to green-light features. The pooled held-out RMSEs were 12.87 and 13.16 mmHg for (**j**) and (**k**), respectively, with corresponding *R*^2^ values of 0.87 and 0.81. The colored lines and markers distinguish the reference SBP values, estimated SBP values, and their corresponding relationships, as indicated in the individual panels.

**Figure 9:**
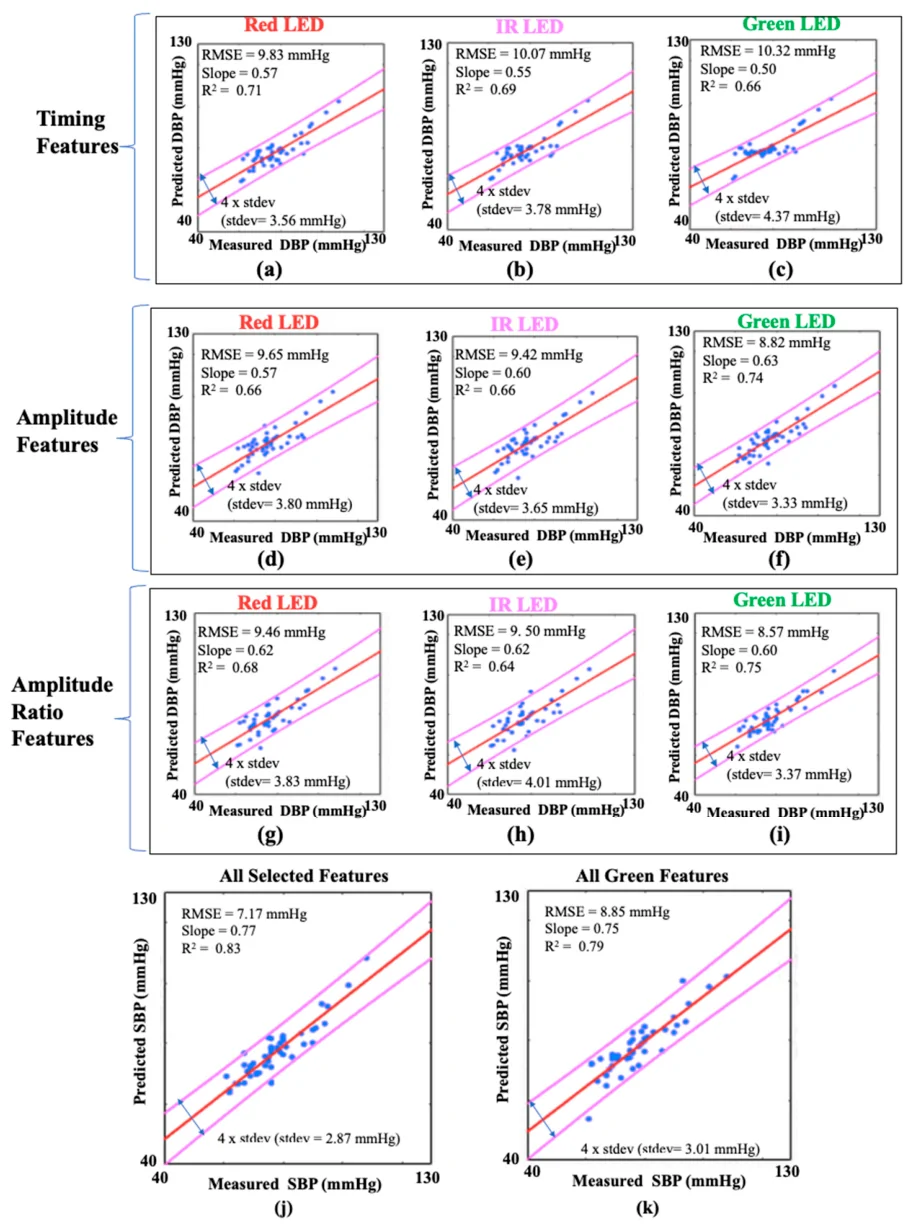
Diastolic blood pressure (DBP) estimation using different PPG feature categories with participant-level ten-fold cross-validation (48 participants). (**a**–**c**) Timing features from red, infrared, and green PPG, respectively; (**d**–**f**) amplitude features from the three wavelengths; and (**g**–**i**) amplitude ratio features from the three wavelengths. (**j**) Features selected from all three wavelengths using only the nine training folds in each iteration (*p* < 0.05). (**k**) Corresponding feature selection restricted to green-light features. The pooled held-out RMSEs were 7.17 and 8.85 mmHg for (**j**) and (**k**), respectively, with corresponding *R*^2^ values of 0.83 and 0.79. The colored lines and markers distinguish the reference DBP values, estimated DBP values, and their corresponding relationships, as indicated in the individual panels.

This procedure was repeated independently for all ten folds; feature selection and regression coefficients were recalculated in each iteration. Each participant received one held-out prediction. Predictions from all ten test folds were pooled to calculate RMSE and *R*^2^ and to construct the measured-versus-estimated BP plots in Figure 8 and Figure 9. We also recorded how many iterations selected each feature; features selected in at least five iterations were designated as frequently selected in Figure 7.

## 3. Results

Figure 7 presents the univariate Pearson correlations, the 30 extracted PPG features listed in Table 3, and cuff-based SBP and DBP measurements across all 48 participants. The numbers adjacent to the bars indicate how many of the ten cross-validation iterations selected each feature using only the nine training folds. In Figure 7a, among the timing features, only *t_SV_* exhibited a statistically significant correlation with SBP. The correlation coefficients for *t_SV_Red_*, *t_SV_IR_*, and *t_SV_Green_* were 0.43, 0.37, and 0.30, respectively. These features were selected in 10, 10, and 5 iterations. Among the amplitude features, *Amp_NV_Green_* and *Amp_DV_Green_* had full-cohort correlations of −0.29 and −0.37 with SBP and were selected in 6 and 10 iterations, respectively. *Amp_SV_Green_* was selected in 3 iterations; the red- and infrared-light amplitude features were not selected. The dicrotic-notch amplitude ratios showed stronger positive correlations with SBP. For *Ratio_SN_*, the correlation coefficients were 0.39, 0.41, and 0.56 for red, infrared, and green PPG, respectively. The corresponding values for *RatioSN*^2^ were 0.40, 0.42, and 0.57. All six features were selected in all ten iterations. By comparison, *Ratio_SD_* and *Ratio_SD_*^2^ showed weaker full-cohort correlations with SBP (*r* < 0.15) and were not selected in any iteration.

Figure 7b presents the corresponding results for DBP. None of the timing features (*t_SV_*, *t_NS_*, and *t_DS_*) was selected in any iteration. Among the amplitude features, *Amp_SV_Green_*, *Amp_NV_Green_*, and *Amp_DV_Green_* were each selected in all ten iterations, whereas the red- and infrared-light amplitude features were not selected. As in the SBP analysis, dicrotic-notch amplitude ratios showed positive correlations with DBP. For *Ratio_SN_*, the correlation coefficients were 0.34, 0.37, and 0.48 for red, infrared, and green PPG, respectively; for *Ratio_SN_*^2^, they were 0.33, 0.38, and 0.51. All six features were selected in all ten iterations. *Ratio_SD_* and *Ratio_SD_*^2^ were not selected in any iteration. Overall, *Ratio_SN_* and *Ratio_SN_*^2^ showed relatively strong full-cohort correlations and were consistently selected within the training folds for both SBP and DBP.

Figure 8 illustrates the estimation performance of different PPG feature categories for systolic blood pressure (SBP). In Figure 8a–c, SBP estimation based on timing features (*t_SV_*, *t_NS_*, and *t_DS_*) from red-light, infrared-light, and green-light PPG yielded root mean square errors (RMSEs) of 17.26, 17.53, and 17.75 mmHg, respectively. In contrast, amplitude features (*Amp_SV_*, *Amp_NV_*, and *Amp_DV_*) (Figure 8d–f) produced substantially higher RMSEs of 32.85, 35.93, and 37.31 mmHg for red, infrared, and green light, respectively.

When using amplitude ratio features (*Ratio_SN_* and *Ratio_SD_*) without squared terms Figure 8g–i, the SBP RMSEs were 16.27 mmHg for red PPG, 16.05 mmHg for infrared PPG, and 13.93 mmHg for green PPG. For Figure 8j, features with training-fold correlation *p* < 0.05 were selected from all three wavelengths separately in each cross-validation iteration. The pooled held-out predictions yielded an RMSE of 12.87 mmHg and an *R*^2^ of 0.87. Restricting this selection procedure to green-light features, Figure 8k yielded an RMSE of 13.16 mmHg and an *R*^2^ of 0.81. These were the lowest SBP RMSEs among the feature sets evaluated. Notably, only Figure 8j and Figure 8k achieved *R*^2^ values greater than 0.8 (0.87 and 0.81, respectively), indicating that carefully selected PPG features provide superior predictive performance for cuffless SBP estimation.

Figure 9 presents the estimation results of diastolic blood pressure (DBP) using different PPG feature categories. In Figure 9a–c, DBP estimation based on timing features from red-light, infrared-light, and green-light PPG achieved root mean square errors (RMSEs) of 9.83, 10.07, and 10.32 mmHg, respectively. For amplitude features (Figure 9d–f), the RMSEs were 9.65 mmHg (red light), 9.42 mmHg (infrared light), and 8.82 mmHg (green light). When using amplitude ratio features without squared terms Figure 9g–i, the DBP RMSEs were 9.46 mmHg for red PPG, 9.50 mmHg for infrared PPG, and 8.57 mmHg for green PPG. For Figure 9j, features with training-fold correlation *p* < 0.05 were selected from all three wavelengths separately in each cross-validation iteration. The pooled held-out predictions yielded an RMSE of 7.17 mmHg and an *R*^2^ of 0.83, the lowest DBP RMSE among the evaluated models. Restricting the selection procedure to green-light features Figure 9k yielded an RMSE of 8.85 mmHg and an *R*^2^ of 0.79. Figure 9j and Figure 9k achieved *R*^2^ values approaching 0.8 (0.83 and 0.79, respectively), also confirming that carefully selected PPG features substantially improve DBP estimation accuracy.

## 4. Discussion

The primary objective of this exploratory study was to compare PPG-derived features from red, infrared, and green light for systolic blood pressure (SBP) and diastolic blood pressure (DBP) estimation. Particular attention was given to whether amplitude ratios derived from identifiable waveform landmarks provide information beyond individual timing or amplitude features. In the present cohort, amplitude ratio features, especially those involving the systolic peak and dicrotic notch, showed stronger associations with cuff-based BP readings than most individual timing and amplitude features.

The present study differs from conventional pulse arrival time (PAT)-based BP estimation methods. PAT is commonly measured as the interval from the ECG R wave to a fiducial point in a peripheral PPG waveform and therefore requires synchronized ECG and PPG recordings [23,24]. Although PAT-based approaches have been extensively investigated for BP estimation, their conventional implementation is less suitable when only a PPG sensor is available. The objective of this study was to explore a PPG-only approach using timing, amplitude, and amplitude ratio features derived from red, infrared, and green PPG signals. Because ECG was not recorded, conventional ECG–PPG PAT could not be calculated or directly benchmarked using the present dataset. Future studies incorporating simultaneous ECG and PPG recordings should compare the proposed PPG-only approach with established PAT-based models in the same participants.

PPG fiducial points have previously been identified using waveform areas, derivatives, and morphological criteria [10,14,20]. In this study, finite-difference derivative estimates were combined with the conventional curvature formula to locate candidate valleys, systolic peaks, dicrotic notches, and diastolic peaks. The contribution of this procedure lies in its application to extracting and comparing features across three PPG wavelengths, rather than in a new mathematical definition of curvature. The preliminary detection-length analysis presented in Figure 3 provides a rationale for the short setting used in this implementation. However, that analysis was based on one separate recording. Moreover, the present study did not compare detected landmarks against manual annotations or directly benchmark the curvature method against a second-derivative detector. Therefore, claims that curvature detection is more accurate, less sensitive to noise, or more reproducible than established methods require further evaluation.

Green-light PPG provided several informative features in this cohort (Figure 7). The full-cohort correlations between green-light *Ratio_SN_* and SBP and DBP were approximately 0.56 and 0.48, respectively. In the training-fold analyses, green-light *Ratio_SN_* and *Ratio_SN_*^2^ were selected in all ten iterations for both outcomes. Green-light selected-feature models yielded cross-validated *R*^2^ values of 0.81 for SBP and 0.79 for DBP (Figure 8k and Figure 9k), compared with 0.87 and 0.83 when selected features from all three wavelengths were combined (Figure 8j and Figure 9j). The amplitudes measured at different wavelengths should not be interpreted as directly comparable measures of blood volume or BP. Each signal reflects a combination of wavelength-dependent absorption and scattering, the vascular tissue sampled, baseline vessel diameter and compliance, and sensor contact. Accordingly, our wavelength comparison concerns the empirical associations and held-out model performance of features extracted from each signal; it does not isolate the contribution of any individual optical or vascular mechanism. Differences in optical penetration and the vascular compartments sampled by different wavelengths may contribute to these observations. Nevertheless, the present data do not isolate the effects of optical absorption, tissue depth, vascular compliance, and sensor contact. The observed advantage of particular green-light features should therefore be interpreted as an empirical finding in this cohort, rather than proof of a single underlying optical mechanism or established superiority across populations.

Among the investigated morphological features, Ratio_SN_ showed a stronger association with both SBP and DBP than Ratio_SD_. Because an amplitude ratio expresses two landmarks relative to one another within a waveform, it may reduce some variation associated with absolute signal amplitude. The weaker performance of Ratio_SD_ may reflect variability in the appearance and detection of the diastolic peak, as well as differences in wave reflection and vascular properties between individuals [25,26,27]. These explanations remain hypotheses that were not directly tested in the present study. The squared *Ratio_SN_* term was included to explore a possible nonlinear association between waveform morphology and BP. Its inclusion in a linear regression model allows a curved relationship with that feature, but does not establish a particular vascular-mechanics model. Because amplitude ratios, their squared terms, and corresponding features from different wavelengths may be correlated, the stability and interpretation of individual regression coefficients also require further examination.

This inter-individual variability limits its value for regression models. Similar patterns were observed in timing features: among *t_SV_*, *t_NS_*, and *t_DS_*, only *t_SV_* showed statistical significance for SBP, reflecting its link to left ventricular contractility. Consequently, the timing features achieved moderate explanatory power (R^2^ ≈ 0.7) for SBP across individual light sources. We further enhanced the regression model by including squared terms of Ratio_SN_, motivated by vascular mechanics models (Hooke’s law, polynomial arterial models, and exponential approximations) [25,26,27] that demonstrate nonlinear relationships between vascular compliance and pressure. Incorporating Ratio_SN_^2^ improved both SBP and DBP estimation accuracy.

**Figure 10:**
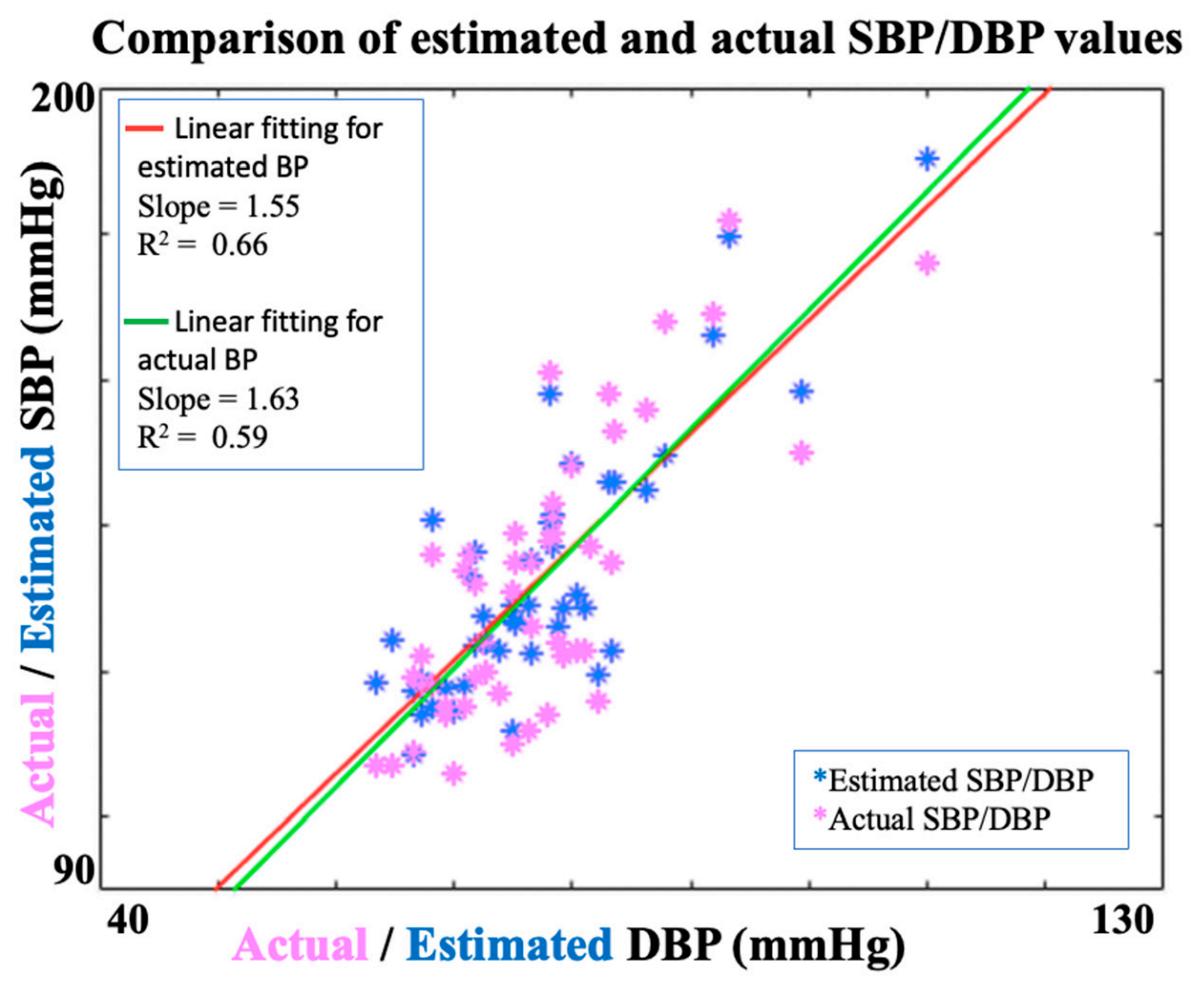
Comparison of the relationships between systolic blood pressure (SBP) and diastolic blood pressure (DBP) in the estimated and cuff-measured datasets. Linear regression yielded slopes of 1.55 (R^2^ = 0.66) for estimated BP and 1.63 (R^2^ = 0.59) for cuff-measured BP. These descriptive similarities characterize the SBP–DBP relationships within the two datasets but do not independently establish agreement between estimated and measured BP values.

Separate regression models were developed for SBP and DBP. Figure 7 shows that several dicrotic-notch amplitude ratio features had positive correlations with both outcomes and were consistently selected within the training folds. Figure 10 compares the relationships between SBP and DBP in the measured and estimated datasets. The corresponding slopes were 1.63 and 1.55, with *R*^2^ values of 0.59 and 0.66, respectively. These descriptive similarities do not independently establish the accuracy of individual BP estimates; prediction errors are reported in Figure 8 and Figure 9. These findings are consistent with the symmetric regression slope of 1.58 reported by Gavish et al. (2008) in 24-h ambulatory blood pressure monitoring (ABPM) studies [28].

The best cross-validated RMSE was 12.87 mmHg for SBP and 7.17 mmHg for DBP relative to the intermittent cuff-based readings. Cuff BP measurement was initiated approximately 2 s after the end of the 90 s PPG recording. Participants maintained the same seated posture, with both arms supported on the chair’s armrests, and were instructed to remain still and refrain from speaking throughout the procedure. Although these conditions were maintained to reduce procedural variability, they do not establish that the physiological state remained unchanged. BP may have varied during PPG acquisition or before and during the subsequent cuff measurement. Because no simultaneous or repeated reference measurements were obtained, the magnitude and direction of error attributable to this temporal mismatch cannot be quantified. Accordingly, the subsequent cuff reading cannot be assumed to represent BP throughout the preceding PPG recording or at every individual cardiac cycle.

The present study also did not constitute standardized clinical validation of BP measurement accuracy. The cohort comprised 48 participants and was not recruited according to the sample-size and predefined BP-distribution requirements of a BHS or AAMI/ESH/ISO validation protocol. Reference BP values were obtained from an automated upper-arm cuff device, rather than auscultatory measurements performed by two qualified observers. These limitations affect both the BP labels used for model development and the interpretation of prediction errors. Participant-level cross-validation evaluates internal prediction performance relative to the available cuff readings; it cannot compensate for limitations in the reference procedure or establish clinical measurement accuracy. Furthermore, RMSE is not interchangeable with the mean difference and standard deviation of paired errors used in standardized BP-device validation. In particular, the substantial SBP error precludes a claim of clinical measurement accuracy based on the present results. The findings should therefore be interpreted as exploratory associations and internally evaluated predictions relative to cuff-based BP readings in this cohort. Improving estimation performance and conducting an independent, prospectively designed study under an appropriate validation framework are necessary before clinical BP measurement claims can be supported.

Another limitation is the relatively small cohort and the variability in participant age. The present study included 48 participants with a mean age of 63.41 ± 14.20 years. Age-related differences in vascular compliance, arterial stiffness, and peripheral hemodynamics may influence PPG waveform morphology and the amplitude ratio features examined in this study. Although participant-level cross-validation was employed to evaluate the proposed estimation models, the limited sample size did not allow reliable assessment of age-dependent feature–BP relationships or model performance across different age groups. Therefore, the generalizability of the observed associations to broader populations remains uncertain. Future studies involving larger and more age-diverse cohorts are needed to investigate the influence of age-related vascular characteristics on multi-wavelength PPG features and BP estimation performance.

Several signal-processing choices also limit interpretation. The 0.5–5 Hz filter may influence PPG waveform morphology and the timing of curvature-derived landmarks. The 50% curvature-peak threshold and fixed 100 ms cross-wavelength matching tolerance were operational choices whose effects on retained cardiac cycles and model performance have not yet been systematically quantified. Future work should compare landmark detection against annotated waveforms, examine sensitivity to filtering and detection parameters, assess correlated predictors and the incremental value of timing features, and test the resulting models in a larger and more diverse cohort. Repeated, time-aligned BP reference measurements and an appropriate clinical validation protocol will be necessary before considering clinical measurement or continuous-monitoring claims.

In addition to further human-subject investigations, future work may incorporate a laboratory-based pulsatile flow phantom to evaluate the relationships between PPG waveform morphology and controlled pressure variations. Such a setup could integrate tissue-mimicking materials with optical absorption and scattering properties resembling those of human tissue, together with compliant vascular structures that approximate the mechanical behavior of peripheral arteries. By systematically varying pulsatile pressure and vascular compliance while obtaining synchronized reference pressure measurements, the effects of these factors on curvature-derived fiducial points and amplitude ratio features could be examined under controlled conditions. This approach would complement human-subject measurements by providing a more controlled environment for investigating the physiological and optical factors influencing multi-wavelength PPG features. However, phantom-based experiments would not replace the need for validation in larger and more diverse human populations.

## 5. Conclusions

This exploratory study investigated cuffless blood pressure (BP) estimation using curvature-derived features from red, infrared, and green PPG signals. The curvature-based procedure was used to identify pulse valleys, systolic peaks, dicrotic notches, and diastolic peaks for the extraction of timing, amplitude, and amplitude ratio features. Among the features examined, amplitude ratios involving the systolic peak and dicrotic notch, particularly Ratio_SN_, showed the strongest associations with cuff-based systolic BP (SBP) and diastolic BP (DBP) readings. Green-light PPG provided several informative features in this cohort, while models that selected features from all three wavelengths within each training fold achieved the lowest reported SBP and DBP RMSEs.

Using participant-level 10-fold cross-validation, the best combined models achieved root mean square errors of 12.87 mmHg for SBP and 7.17 mmHg for DBP, with corresponding R^2^ values of 0.87 and 0.83. These findings support further investigation of multi-wavelength PPG morphology and amplitude ratio features for BP estimation. However, the SBP error is substantial, and the present study does not establish clinical measurement accuracy or continuous BP tracking. The cohort comprised 48 participants, and each 90 s PPG recording was associated with a single cuff BP measurement obtained afterward. Future studies should evaluate feature-detection and model stability in larger, age-diverse cohorts, investigate the effects of vascular mechanical and tissue optical properties through controlled phantom-based experiments, and employ repeated, closely time-aligned BP reference measurements under appropriate clinical validation protocols.

## Figures and Tables

**Table 1:** Publicly available PPG-based blood pressure databases.

Dataset Name	Population Size	Wavelengths Used (nm)	Sampling Rate	Notes
MIMIC-II/III/IV (PhysioNet)	>60,000 ICU records (subset with PPG + ABP available)	Red (~660), IR (~940)	125 Hz	Very large ICU database, invasive arterial BP reference.
PPG-BP Database (UCI/Kaggle)	219 subjects	Red (660), IR (940)	125 Hz	Non-invasive cuff BP, widely used for machine learning studies.
BIDMC PPG & Respiration (PhysioNet)	53 ICU patients	Red (660), IR (940)	125 Hz	Includes invasive ABP waveform, smaller but high-quality dataset.

**Table 2:** Demographic characteristics of systolic and diastolic blood pressure among the 48 subjects.

Group	N	SBP (Mean ± SD)	DBP (Mean ± SD)	SBP > 140	DBP > 90	Both SBP > 140 & DBP > 90
Female	25	139.99 ± 19.14	83.54 ± 10.56	9	2	2
Male	23	136.21 ± 19.55	80.24 ± 11.79	6	3	2
Overall	48	138.18 ± 19.23	81.96 ± 11.17	15	5	4

**Table 3:** Definitions of the 30 candidate PPG features used for blood pressure estimation.

Feature Number	Feature Type	Feature Name	Description
1	Timings	t_SV_Red_	Time interval of systolic peak to valley in red-light PPG
2	Timings	t_SV_IR_	Time interval of systolic peak to valley in infrared-light PPG
3	Timings	t_SV_Green_	Time interval of systolic peak to valley in green-light PPG
4	Timings	t_NS_Red_	Time interval of dicrotic notch to systolic peak in red-light PPG
5	Timings	t_NS_IR_	Time interval of dicrotic notch to systolic peak in infrared-light PPG
6	Timings	t_NS_Green_	Time interval of dicrotic notch to systolic peak in green-light PPG
7	Timings	t_DS_Red_	Time interval of diastolic peak to systolic peak in red-light PPG
8	Timings	t_DS_IR_	Time interval of diastolic peak to systolic peak in infrared-light PPG
9	Timings	t_DS_Green_	Time interval of diastolic peak to systolic peak in green-light PPG
10	Amplitudes	Amp_SV_Red_	Amplitude between systolic peak and valley in red-light PPG
11	Amplitudes	Amp_SV_IR_	Amplitude between systolic peak and valley in infrared-light PPG
12	Amplitudes	Amv_SV_Green_	Amplitude between systolic peak and valley in green-light PPG
13	Amplitudes	Amp_NV_Red_	Amplitude between dicrotic notch and valley in red-light PPG
14	Amplitudes	Amp_NV_IR_	Amplitude between systolic peak and valley in infrared-light PPG
15	Amplitudes	Amp_NV_Green_	Amplitude between systolic peak and valley in green-light PPG
16	Amplitudes	Amp_DV_Red_	Amplitude between diastolic peak and valley in red-light PPG
17	Amplitudes	Amp_DV_IR_	Amplitude between diastolic peak and valley in infrared-light PPG
18	Amplitudes	Amp_DV_Green_	Amplitude between diastolic peak and valley in green-light PPG
19	Amplitude Ratios	Ratio_SN_Red_	Amplitude ratio of systolic peak to dicrotic notch in red-light PPG
20	Amplitude Ratios	Ratio_SN_IR_	Amplitude ratio of systolic peak to dicrotic notch in infrared-light PPG
21	Amplitude Ratios	Ratio_SN_Green_	Amplitude ratio of systolic peak to dicrotic notch in green-light PPG
22	Amplitude Ratios	Ratio_SD_Red_	Amplitude ratio of systolic peak to diastolic peak in red-light PPG
23	Amplitude Ratios	Ratio_SD_IR_	Amplitude ratio of systolic peak to diastolic peak in infrared-light PPG
24	Amplitude Ratios	Ratio_SD_Green_	Amplitude ratio of systolic peak to diastolic peak in green-light PPG
25	Amplitude Ratios	Ratio_SN_Red_^2	The squared amplitude ratio of systolic peak to dicrotic notch in red-light PPG
26	Amplitude Ratios	Ratio_SN_IR_^2	The squared amplitude ratio of systolic peak to dicrotic notch in infrared-light PPG
27	Amplitude Ratios	Ratio_SN_Green_^2	The squared amplitude ratio of systolic peak to dicrotic notch in green-light PPG
28	Amplitude Ratios	Ratio_SD_Red_^2	The squared amplitude ratio of systolic peak to diastolic peak in red-light PPG
29	Amplitude Ratios	Ratio_SD_IR_^2	The squared amplitude ratio of systolic peak to diastolic peak in infrared-light PPG
30	Amplitude Ratios	Ratio_SD_Green_^2	The squared amplitude ratio of systolic peak to diastolic peak in green-light PPG

## Data Availability

The data presented in this study are available from the corresponding author upon reasonable request. The data are not publicly available due to privacy and ethical considerations related to human participant data.
